# Poly(vinyl butyral) Composites with Different Silicate or Silica Dispersions

**DOI:** 10.3390/polym18040476

**Published:** 2026-02-13

**Authors:** Vasilis Nikitakos, Christophoros Razos, Athanasios D. Porfyris, Constantine D. Papaspyrides, Konstantinos G. Beltsios

**Affiliations:** 1 Laboratory of Polymer Technology, School of Chemical Engineering, Zographou Campus, National Technical University of Athens, 15780 Athens, Greece; vnikitakos@mail.ntua.gr (V.N.); xristoforos200221@gmail.com (C.R.); kp@cs.ntua.gr (C.D.P.); 2 Research Lab of Advanced, Composites, Nanomaterials and Nanotechnology (R-NanoLab), School of Chemical Engineering, Zographou Campus, National Technical University of Athens, 15772 Athens, Greece; 3 Laboratory of Materials Science & Engineering, School of Chemical Engineering, Zographou Campus, National Technical University of Athens, 15780 Athens, Greece

**Keywords:** PVB, silicate, silica, composite, glass, upcycling, laminated, particulate

## Abstract

Poly(vinyl butyral) (PVB) displays exceptional adhesion to glass surfaces and high transparency, serving as the dominant interlayer material in laminated glass composites. This study systematically investigates PVB particulate composites, focusing on the interactions between a plasticized PVB matrix and silicate or silica dispersions as reinforcements. PVB composites reinforced with glass flakes, glass fibers, and fumed silica at loadings of 2, 5, and 8 vol% were produced and characterized. Optical microscopy and thermogravimetric analysis were employed to evaluate filler incorporation and dispersion under melt mixing conditions representative of industrial extrusion. Transparency measurements assessed the optical clarity of the composites, while ATR-FTIR was used to identify chemical interactions between PVB and the fillers. Regarding mechanical performance, fumed silica increased tensile strength up to 29 MPa and reduced displacement at fracture by 120%, while high-aspect-ratio flakes and silane-treated fibers only significantly increased composite stiffness. Impact resistance was additionally evaluated, revealing a significant enhancement upon the addition of fibrous reinforcements, especially when silane-treated fibers were used. Fumed silica increased the thermal stability of PVB by 7 °C and reduced water uptake to approximately 4.5%, in contrast to glass flakes, which increased water absorption reaching up to 8–11%. Lastly, the processability of composites was monitored, showing a progressive decrease with increasing filler content for all reinforcements. Overall, this work provides a comprehensive assessment of PVB–silicate/silica interfacial interactions and highlights the design of PVB composites suitable for advanced applications or the upcycling of secondary recycled PVB grades.

## 1. Introduction

PVB (polyvinyl butyral) is a thermoplastic specialty polymer mostly used in glass laminate composites due to its strong adhesion to polar substrates. It is produced by acid-catalyzed reaction of poly(vinyl alcohol) (PVA) with butyraldehyde (BA), resulting in a random and amorphous terpolymer, composed primarily of 70–85% vinyl butyral (VB), 10–25% unreacted vinyl alcohol (VA), and 1–5% residual vinyl acetate (VAc) [1,2,3]. Neat PVB exhibits a glass transition temperature (*T*_g_) in the range of 65–75 °C, thus being a rigid polymer at room temperature. Commercially, it is available primarily in plasticized form, typically containing 20–45% *w*/*w* glycol esters, adipates, or sebacates to achieve the required processability and flexibility for its applications [4,5,6]. The strong adhesion between PVB and glass originates from hydrogen or covalent bonding between the hydroxyl groups of the polymer and the silanol groups present on the glass surface [7]. Strong bonding performance to glass and exceptional transparency establish PVB as an excellent fit for an interlayer material between two glass sheets forming laminated glass. This composite material combines the mechanical and optical advantages of glass with the viscoelastic nature of plasticized PVB, allowing for energy absorption, maintaining adhesion to fractured glass, and preventing dangerous shards from scattering in the event of an accident. Due to these properties, PVB is the most widely used interlayer material in laminated safety glass for automotive and architectural applications, along with secondary uses in photovoltaic encapsulants, coatings, and adhesives [8].

The adhesion performance of PVB in laminated composites is largely controlled by its interfacial interactions with the glass substrate, which remains not fully elucidated. Optimal adhesion to glass is achieved by adjusting the percent of unreacted hydroxyl groups (–OH) to 17–23% after the reaction process [4,6,9]. While insufficient adhesion reduces glass retention during impact, excessively strong adhesion (increased vinyl alcohol content) diminishes impact resistance by enabling glass-initiated cracks to propagate through the interlayer and lower resistance to projectile penetration [10,11]. Therefore, proper control of interfacial strength can be modulated via the film composition, e.g., content of polyvinyl alcohol and the presence of adhesion-regulating additives [12]. Furthermore, environmental factors like temperature and humidity also affect adhesion to glass and consequent properties such as post-crack behavior, impact resistance and crack propagation [13,14]. Moisture can disrupt the interaction between PVB and the silanol groups, resulting in lower bonding, as indicated by reduced Pummel test values [15,16,17,18]. Additionally, recent experimental and modeling studies have highlighted the effect of rate- and temperature-dependent and viscoelastastic mechanical response of PVB interlayers, further affecting the overall performance in laminated glass systems [19,20,21,22,23,24,25]. Modeling studies have used finite element and viscoelastic modeling frameworks to simulate the impact and deformation behavior of laminated glass with PVB interlayers, focusing mainly on structural response and ignoring the effect of other parameters such humidity or interfacial chemical interactions [22,26].

Furthermore, the surface roughness of glass has a significant effect, as the rougher air side promotes mechanical interlocking and thus stronger adhesion compared to the smoother tin side, which contains Sn^2+^/Sn^4+^ ions and exhibits slightly lower adhesion [27]. The literature suggests that alkanoate salts can hinder adhesion by competing with hydroxyls for bonding sites on the glass surface [11,12]. Tupý et al. found that the addition of magnesium acetate or oligo-functional organic acids during extrusion activated hydroxyl groups in the PVB chains, suggesting that these variations could be essential to ensure the required adhesive strength in the final product [11]. Previous studies in PVB laminated glass have focused exclusively on the interaction of PVB with glass sheets, rather than addressing more holistically its interaction with silica or different silicate substrates, which is the focus of the present work.

Examining different delamination methods also provides valuable insight into the interfacial properties of PVB–glass systems. Delamination approaches include both physical and chemical treatment. Notably for the latter, the interface can be disrupted by introducing an acid solution or base solutions, as documented by various patents and literature references [28,29,30,31,32]. However, strong bases (e.g., KOH or NaOH) or alcoholates can saponify the acetate groups in PVB, thereby modifying its chemical structure and degrading its mechanical performance [33]. Separation can also be achieved using an aqueous medium in the presence of a nonionic surfactant [29,34,35]. Nevertheless, the incorporation of surfactants alone is insufficient to obtain satisfactory delamination and a de-mixing phenomenon can be observed in the film [27,35,36,37]. Modeling studies have employed cohesive-zone models to calibrate the interfacial fracture energy and cohesive strength of PVB–glass interfaces, providing valuable insight into delamination mechanisms under quasi-static loading [38,39]. Nevertheless, the delamination studies so far also focus only on the delamination or mechanical behavior and provide limited insight on the underlying mechanisms of PVB–glass adhesion, which are addressed herein.

Beyond the commercially driven research on laminated composites, the study of a particulate PVB composite with silicate or silica reinforcement, as explored in this work, can also enlighten the interactions of PVB and the silanol groups of glass. Regarding the existing research on this field, Nguyen and Berg showed that increasing vinyl alcohol (VA) content increases adhesion of PVB to glass surfaces by measuring uniaxial tensile stress in PVB with embedded glass beads [40]. Similarly, Miller and Berg have examined how the different types of silane coupling agents can influence the adhesion performance of glass beads within the PVB matrix via uniaxial tensile stress until interfacial failure occurred [41]. In addition, the development of PVB reinforced with glass wastes from flat glass processing was studied by Gorokhovsky et al. and the developed composites demonstrated satisfactory tensile strength and stiffness, highlighting the potential repurposing of glass processing waste into sustainable, structurally useful composites [42]. Complementary, Scharfe et al. have investigated the optical and mechanical properties of particulate composites prepared with Micro Glass Flakes (MGFs), reporting enhanced stiffness and minimal haze [43]. More studies of particulate PVB composites prepared via solution casting showed that the addition of SiO_2_ improved stiffness and thermal stability of PVB, attributed mainly to restricted chain mobility and filler–matrix interactions [44,45]. To date, only a limited number of studies have examined this type of interfacial interaction, and they mainly focus solely on one reinforcement or the mechanical behavior.

This study on particulate PVB composites aims to fill a significant knowledge gap regarding adhesion mechanisms by providing a systematic experimental comparison of multiple silicate and silica reinforcements with distinct morphologies (glass flakes, glass fibers, and fumed silica) at various filler loadings (2–8% *v*/*v*) in a commercially relevant plasticized PVB matrix. The composites were prepared using an internal mixer, representative of industrial processing conditions in contrast to solution casting methodologies. The effects of the different interface interactions are studied, employing a range of characterization techniques, including ATR-FTIR, optical microscopy, light transmittance, thermal analysis (TGA-DSC), mechanical testing (tensile and impact strength), melt flow rate analysis, parallel-plate rheometry and water absorption testing. In this paper, advancing from previous studies, interactions of PVB are more closely related to macroscopic properties and potential applications are suggested including mechanically toughened laminated glass, thermally resistant coatings, PVB films with improved water barrier properties for photovoltaic encapsulants, and advanced 3D-printable PVB materials. This proposed upcycling route targets also the valorization of secondary recycled PVB grades obtained after delamination, which might remain unexploited, thus contributing to circular economy. To the best of our knowledge, there is currently no other comprehensive study examining the effect of silicate or silica reinforcements within a commercial polyvinyl butyral (PVB) matrix.

## 2. Materials and Methods

### 2.1. Materials

Plasticized PVB sheets (PVBR-B) were supplied by Eastman Chemical Company (Kingsport, TN, USA) under the trade name Saflex RB41. PVBR-B containedapproximately 31% *w*/*w* triethylene glycol bis(2-ethylhexanoate) (3GO) plasticizer and was used as the polymeric matrix for all the composites. Unplasticized PVB (u-PVB) was supplied by American Polymer Standards Corporation (Mentor, OH, USA) and pure 3GO plasticizer was supplied by Eastman Chemical Company (Kingsport, TN, USA). Silicate particles of varying dimensions and morphologies were used as fillers, as shown in Table 1. Fumed silica under the trade name Aerosil 200 was produced by Evonik Industries AG (Essen, Germany). 3-Aminopropyldiethoxymethylsilane (APDEMS), produced by Tokyo Chemical Industry (Tokyo, Japan), was used as a silane coupling agent for the treatment of glass fibers. Glass fibers (S-Gfiber) functionalized with APDEMS were also investigated on their interfacial adhesion to the PVB matrix.

### 2.2. Preparation of Samples

A Banbury-type internal mixer T300B (Brabender, Duisburg, Germany) was used for mixing the 3GO plasticizer with u-PVB and the incorporation of the various fillers with the reference polymer matrix (PVBR-B). Dried u-PVB batches were introduced into a preheated chamber (150 °C) and processed for 5 min at a constant screw speed of 40 rpm, using various weight concentrations of plasticizer (10, 20, 30, or 40% *w*/*w*). Identical processing conditions were also applied for the case of particulate composites, while the fillers were incorporated at various volume contents of 2, 5, and 8 vol%. The filler content in the composites is reported in vol% (volume percent) because composite mechanics and theoretical models are formulated in terms of constituent volume contents, which govern load sharing and effective mechanical properties. The processing conditions of mixing were selected based on previous findings, which identified them as optimal for efficient mixing while minimizing polymer degradation [36,46]. Additionally, 0.3% *w*/*w* of a multifunctional antioxidant Irganox 565 (Ludwigshafen, Germany) was incorporated into each batch to minimize thermo-oxidative degradation of PVB during processing [36]. Once mixing was completed, the material was removed from the chamber, left to cool and dried under vacuum before undergoing further analysis.

Functionalization with mono-aminosilane agent (APDEMS) on glass fiber was employed to obtain a better interface with PVB matrix and further study the corresponding influence on the adhesion strength and properties [41]. A silane concentration of 0.5% *w*/*w* in pure water was used and it was left for 60 min to hydrolyze. Subsequently, the glass fibers (Gfiber) were added to the solution and left for 90 min to complete the reaction with the silane coupling agent. Finally, they were removed, washed with water, and dried at 60 °C overnight.

### 2.3. Methods

The morphology and surface of PVB composites were examined by optical microscope Examet Union 82160 (Unitron, St. John’s, NL, Canada) equipped with a Sony CCD-IRIS SSC-C370P (Tokyo, Japan) digital camera. The composite specimens were rapidly frozen using liquid nitrogen and then cryo-cut into cross-sections using pliers to obtain smooth, undistorted surfaces for microscopic analysis and observation of the filler dispersion.

Transparency (light transmission) of each composite sample in the form of films of 1 mm thickness was recorded using a calibrated handheld Dr. Lange Spectro-Color LMG 183 spectrophotometer (Berlin, Germany). Measurements were conducted in triplicate at multiple points across each film to ensure reliability of the extracted data.

ATR (Attenuated Total Reflection) FT-IR spectroscopy measurements were performed on a Bruker AII ATR Spectrometer (Bruker Corporation, Billerica, MA, USA) from 400 to 4000 cm^−1^ with a 4 cm^−1^ resolution using a diamond crystal. 32 co-added spectra were taken for each measurement. All samples were in the form of thin films of ca. 300 μm thickness prepared via compression molding.

Thermal stability of the PVB samples was determined by thermogravimetric analysis (TGA) in a Mettler Toledo TGA/DSC HT 1 (Mettler-Toledo International Inc., Greifensee, Switzerland) apparatus. The samples were analyzed through dynamic heating from 30 to 600 °C at a rate of 10 °C/min, under controlled nitrogen flow (10 mL/min). Plasticizer evaporation temperature (*T*_d1_) and PVB decomposition temperature (*T*_d2_) were determined from the peaks of the first derivative curve (DTG) corresponding to the respective maximum rates of mass loss. In addition, residual weight at 600 °C (% *w*/*w*) was calculated to assess the effective filler incorporation. Triplicate measurements were obtained for the accurate assessment of the uniformity of composite PVB samples and the averaged data values refined by the standard deviation were determined.

Differential Scanning Calorimetry (DSC) measurements were conducted in a Mettler DSC 1 (Mettler-Toledo International Inc., Greifensee, Switzerland) module. A heating–cooling–heating cycle from −20 to 210 °C at a heating (cooling) rate of 10 (−10) °C min^−1^ was employed, to erase the thermal history of the material and impose identical cooling/crystallization conditions between the different samples. Nitrogen flow was controlled at 20 mL/min. From the received curves the glass transition temperatures (*T*_g_) of the plasticized PVB samples were determined.

Uniaxial tensile measurements were conducted in accordance with ISO 527-3 on an Instron 4466 universal testing machine (Instron Co., Norwood, MA, USA) equipped with a 10 kN load cell [47]. The tests were run at a grip separation (displacement) rate of 50 mm min^−1^ corresponding to a normalized displacement rate of approximately 1.3 × 10^−2^ s^−1^ and at a controlled temperature of 21 ± 2 °C, representative of room temperature [20,24]. The samples were preconditioned at the testing temperature and at 60 ± 5% relative humidity for 24 h before the measurements. For each formulation, 5 dumbbell Type 5 specimens were prepared by compression molding at 150 °C and ca. 15 MPa. Sandpaper was applied to the ends of the specimens to prevent slippage during testing and to ensure reliable measurements. Normalized displacement (ΔL*) was calculated as the ratio of crosshead displacement (ΔL) to the initial grip-to-grip distance (L_0_) inserted by the user. Normalized displacement should be interpreted carefully when compared with strain values reported in the literature which are obtained via extensometers or optical methods. Additionally, variations in specimen preparation, including thickness uniformity may introduce minor inconsistencies in the measured mechanical response. All tests were performed under controlled laboratory conditions and at a fixed crosshead speed to ensure consistent comparison between formulations, since plasticized PVB is sensitive to specific conditions such as temperature, humidity and applied deformation rate. Stress–normalized displacement diagrams were constructed from the experimental data, from which engineering tensile strength at fracture (*σ*_max_) and normalized displacement at fracture (ΔL*_max_) were also obtained. In the evaluation of the mechanical results, the Secant Modulus (*E*) was also calculated (Equation (1)) as the ratio of the corresponding nominal stress to the designated normalized displacement within the range of 0–0.1. The Secant Modulus was selected because it can be calculated with reasonable accuracy and helps to avoid potential misinterpretations or errors in the initial deformation zone, which may arise from the viscoelastic nature of PVB. This parameter was used in relation to Young’s modulus and can serve as a comparative measure of the stiffness among the different composite formulations, but it does not directly correspond to the material’s fundamental elastic constants.

(1) $$E_{s}=\frac{\Delta \sigma }{\Delta \varepsilon }$$

Izod impact strength tests according to ISO180 were executed and the samples were prepared by injection molding at 150 °C in an Arburg Allrounder 370C machine (Losburg, Germany) [48]. Regarding the impact tests, 4 unnotched specimens of 80 × 10 × 4 mm^3^ per formulation were measured in an Instron Wolpert PW5 apparatus (Norwood, MA, USA) and the determined impact strength (α_iu_, in kJ/m^2^) was calculated according to Equation (2), where E is the absorbed energy during impact in J, h is the width and b is the thickness of each specimen in mm.

(2) $$a_{iu}=\frac{E}{h\times b}\;10^{3}$$

Water absorption tests were conducted according to ASTM D570 [49]. The specimens were first dried at 50 ± 3 °C for 24 h until constant weight to obtain their initial mass (M_1_), then immersed in a water bath at room temperature until equilibrium was reached. After each immersion period, the samples were removed, gently wiped with a clean, dry cloth to remove surface water, weighed immediately (wet mass, M_2_) under analytical scale, and then returned to the bath. Two replicates for each specimen were weighed regularly until a plateau was reached (20 days → 480 h). The percentage of water absorption versus square root of time (hours) was plotted to compute the coefficient of diffusion. The coefficient of diffusion (D) was evaluated using Equation (3) based on Fick’s second law:

(3) $$D={\pi \left(\frac{k\delta }{{4Μ}_{\infty }}\right)}^{2}$$

where k is the slope of the initial linear portion of the M(t) vs. √t curve, ${Μ}_{\infty }$ is the maximum mass gain and δ is the sample thickness (1 mm).

Melt flow rate (MFR) measurements were performed in a Kayeness Dynisco 4004 (Dynisco Europe GmbH, Heilbronn, Germany) melt indexer at 190 °C, with a load of 2.16 kg for all the prepared PVB composite samples according to ASTM D1238-10 to estimate melt rheology in terms of processability [50]. All the samples were dried at 60 °C for 4 h prior to each measurement to exclude plasticization effect of water and measurements were performed in duplicates [51].

Viscoelastic properties of disk-shaped composite samples (25 mm diameter and 1 mm thickness) were characterized using a Thermo HAAKE MARS 40 rotational rheometer (Thermo Fisher Scientific Inc., Waltham, MA, USA) equipped with a 25 mm parallel-plate geometry. Oscillatory shear (strain-controlled) measurements were performed at a typical reprocessing temperature (T = 150 °C). Frequency sweep tests were conducted over the range of 0.01–100 Hz at a fixed strain of 1%, previously confirmed to be within the linear viscoelastic region. The loss factor (tan δ) and complex viscosity (η*) were evaluated as a function of frequency. Proper contact between the sample and the plates was ensured by applying a constant normal force of 10 N.

## 3. Results and Discussion

### 3.1. Morphology of PVB Composites

**Figure 1:**
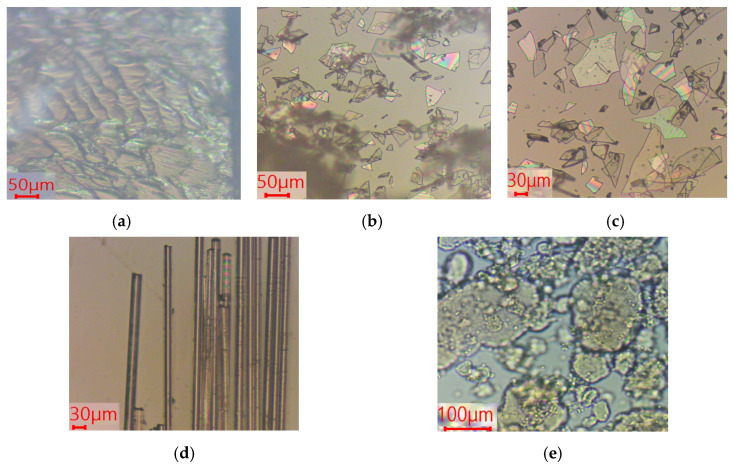
Images of the fillers from optical microscopy (×10): (**a**) PVBR-B in cross section; (**b**) GF-C; (**c**) GF-E; (**d**) glass fibers; (**e**) fumed silica nanoparticles. The color variations are due to the optical microscopy imaging conditions and do not reflect compositional differences.

The morphology, orientation and chemical interactions of the silicate and silica reinforcements with PVB matrix were investigated by optical microscopy, transparency measurements, and FTIR spectroscopy. Cross-sections of composite samples were observed in the light optical microscope (Figure 1). Accordingly, the glass flakes exhibited a flat, sheet-like morphology. GF-C sample (*d*_50_ = 27–32 μm) showed smaller particle sizes compared to GF-E (*d*_50_ = 90 μm) (Figure 1b,c). Some smaller shards observed in GF-E are fragments of larger parts. On the other hand, glass fibers are much longer, but exhibited lower width compared to glass flakes (Figure 1d). Fumed silica corresponded to an agglomerated structure composed of amorphous nanoparticles (Figure 1e).

**Figure 2:**
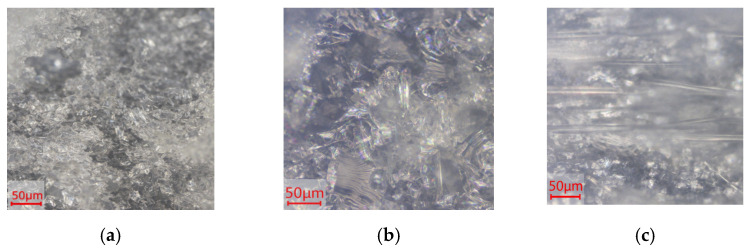
Cross-sectional optical microscopy images (10× magnification) of the material surfaces: (**a**) GF-C (8 vol%); (**b**) GF-E (8 vol%); (**c**) Gfiber (8 vol%).

Turning to the silicate composites and comparing them with pure PVBR-B images, it can be highlighted that the reinforcements are uniformly dispersed and embedded within the PVB matrix (Figure 2a–c), exhibiting random distribution and orientation throughout the polymeric phase. Figure 2 provides images of the composite materials with the same volume content (8 vol%) using glass flakes with different mean diameters (GF-C and GF-E) and also glass fibers, which exhibit greater length. Random dispersion of the glass flakes and fibers was achieved, without preferential orientation and/or agglomeration being observed. The latter comprises an essential factor for maintaining uniform mechanical strength, optical clarity, and overall material consistency. In the case of fumed silica, adequate mixing is assumed to be achieved, as the particles are at the nanoscale, making dispersion easier. Melt mixing in the internal mixer did not induce any specific alignment of the reinforcing components, resulting in a random filler orientation.

**Figure 3:**
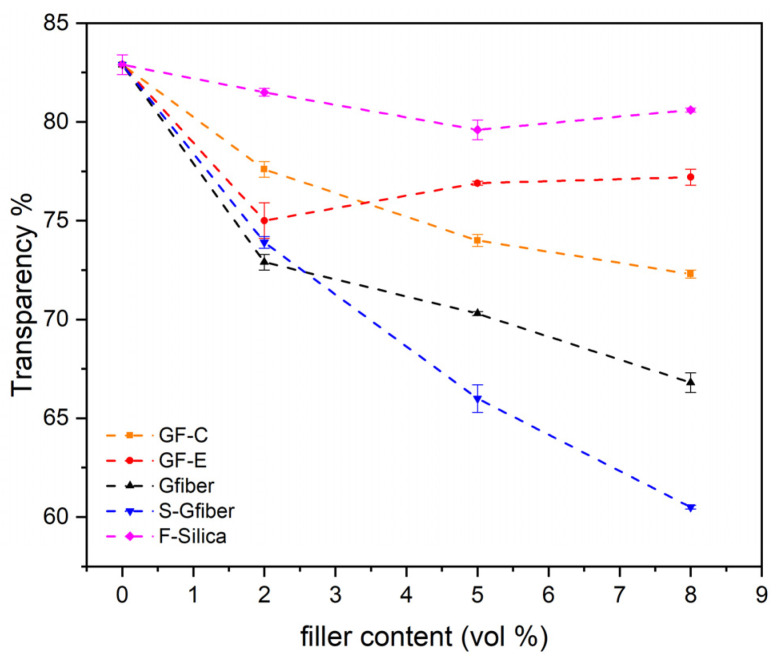
Transparency of PVB composites in correlation with filler content (vol%).

Transparency is a key factor influencing the performance of the composites and their potential applications. As Figure 3 suggests, the incorporation of GF-C reinforcement led to a stronger decrease in transparency compared to GF-E, as it introduced a greater number of scattering centers within the PVB matrix. GF-C with smaller flake size can contain more centers for scattering in the same area, thus increasing haze. This can also be attributed to the higher number of flakes and their random orientation, which introduces multiple light–matrix interfaces and enhances internal scattering. Consequently, lower flake size led to increased optical losses and reduced transparency compared to composites with larger flakes. Larger glass particles or flakes generated fewer interfaces per unit mass, and consequently lower overall scattering. Adding to this, chopped glass fibers created extensive interfacial areas that refract and redirect light in multiple directions, further diminishing transparency. Functionalized glass fibers (S-Gfiber) exhibited even greater light scattering, as the silane coupling treatment enhances interfacial bonding with the PVB matrix and eliminates voids yet increases the refractive index contrast between the two phases. Consequently, silane treatment created a sharper or more defined optical interface between the PVB and the glass fiber, resulting in an optically mismatched boundary increasing haze. Based on Keller et al.’s patent, silane coupling agent was carefully selected to match the refractive index of fumed silica and PVB, to enhance mechanical performance while maintaining high optical transparency [52]. Finally, fumed silica can be added in substantial amounts (even at high contents) without compromising clarity, since it consists of ultra-fine nanoparticles (3–50 nm diameter), able to be homogeneously incorporated in PVB without agglomeration [53,54]. Transparency measurements exhibited very low standard deviation across triplicate measurements, indicating high reproducibility and homogeneity of the composite films.

**Figure 4:**
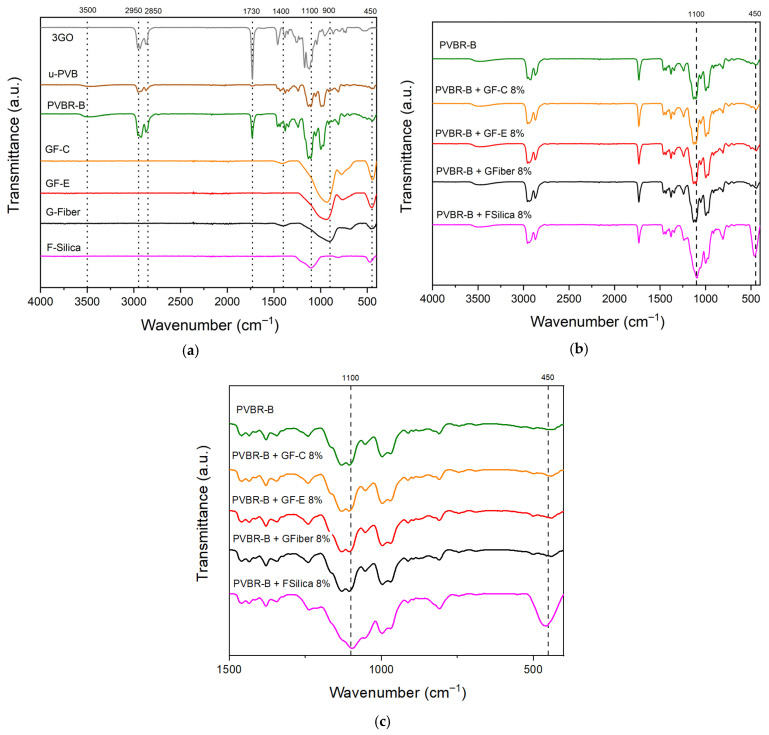
FTIR spectra of (**a**) the initial materials (3GO, u-PVB, PVBR-B, GF-C, GF-E, GFiber, F-Silica); (**b**) PVB Composites at 8 vol%; (**c**) Magnification of the 1500–500 cm^−1^ IR region for (**b**).

The chemical structure of the composites was examined via ATR-FTIR spectroscopy, providing meaningful remarks for the chemical interactions between the PVB matrix and silicate particles. Figure 4a presents the FTIR spectra for the initial reference materials, showing the main peaks of the chemical structures of unplasticized PVB, plasticized PVB, plasticizer, and the various amorphous silicates used. Both u-PVB and PVBR-B exhibit the characteristic peaks of the terpolymer structure. Specifically, the peaks at 1000 and 1100 cm^−1^ correspond to the C–O–C stretching vibrations of the butyraldehyde groups (vinyl butyral content). The broad band observed around 3500 cm^−1^ (≈3200–3600 cm^−1^) is attributed to the O–H stretching vibrations of the vinyl alcohol units. In addition, the peak at 1730 cm^−1^ corresponds to the C=O stretching vibration of carbonyl groups, confirming the presence of ester functionalities in the polymer structure. The most pronounced difference in plasticized and unplasticized sample is clear in intensity at 1730 cm^−1^ and 1100 cm^−1^ due to the presence of plasticizer that corresponds to increased carbonyl content (C=O) and ether bonds (C–O–C stretching vibrations) from triethylene glycol chain accordingly. Also, the C–H symmetric and asymmetric stretching vibrations of methylene and methyl groups (–CH_2_–, –CH_3_) are observed in the 2950–2850 cm^−1^ region, visible in the polymeric samples (u-PVB and PVBR-B) and plasticizer, but foreseeably absent in the inorganic fillers [5,7]. All glass fillers GF-C, GF-E and Gfiber—share the same peak at 900 cm^−1^ which corresponds to Si–O stretching of non-bridging oxygens (NBOs) created by network modifiers (Na^+^, Ca^2+,^ etc.). This is a typical characteristic of glass materials such as E-glass, C-glass, ECR glass or soda-lime glass, whose chemical composition contains various oxides (such as CaO, MgO, Na_2_O, etc.) [55]. In contrast, the FTIR spectrum of fumed silica does not include this peak, since it consists of pure SiO_2_ (>99.8%) and therefore the peak at 1100 cm^−1^ corresponding to asymmetric stretching vibration of Si–O–Si bonds, which are more distinct compared to the other silicate materials. Lastly, the peak at 450 cm^−1^ is present for every silicate material since it is a typical peak corresponding to the bending vibration of the Si–O–Si linkage in the silicate tetrahedral network [56].

On the other hand, Figure 4b displays the ATR-FTIR spectra for composite formulations at 8 vol%, where the spectral changes relative to pure PVBR-B are expected to be the most pronounced and apparent. PVBR-B serves as the reference, representing the characteristic absorption bands of the polymeric matrix. In the composites containing GF-C, GF-E, and Gfiber, no spectral alterations were observed compared to pure PVBR-B. Furthermore, the absence of silicate-related peaks, suggests that only weak chemical interactions may have occurred at the filler–matrix interface. This could be attributed either to the possible overlapping of characteristic peaks or to a uniform dispersion of the fillers within the matrix, making them undetectable in the bulk material. The latter could be attributed to the limited penetration depth of ATR-FTIR, which probes only a very thin surface layer (approximately 0.5–5 μm) [57]. Therefore, interfacial chemical interactions occurring on the surface of the flakes or fibers were not detected and were only observed in the case of fumed silica (strong peak at ca. 450 cm^−1^). Nevertheless, the peak at 3500 cm^−1^ remains constant in all cases, indicating that the outer layer of the prepared film can provide available hydroxyl groups for lamination with glass sheets. This is particularly noteworthy in the case of fumed silica, where chemical interactions were severely pronounced, possibly due to better dispersion in the nanoscale. This indicates that the material retains its potential for use in composite laminated glass, despite the presence of interfacial chemical interactions within the sample. Particular attention should be directed to the composite incorporating fumed silica, which exhibited pronounced spectral deviations relative to pure PVBR-B and the other composites. These alterations are likely attributed to enhanced interfacial interactions and a more uniform dispersion of the nanoscale filler within the polymeric matrix. An apparent increase, sharpening, or broadening of the band around 1050–1100 cm^−1^ is observed, corresponding to Si–O–Si stretching vibrations. This behavior indicates that the material becomes enriched in siloxane-like linkages because of interfacial interactions. The vinyl alcohol units of PVB contain free –OH groups capable of forming hydrogen bonds with surface Si–OH groups. Upon heating during processing, condensation reactions may occur, leading to the formation of Si–O–C bonds and local reinforcement of the siloxane network [7]. The increase in Si–O–C linkages between the hydroxyl groups of PVBR-B and the surface silanols of fumed silica and silicates signifies stronger chemical interactions and improved interfacial adhesion within this composite system [5,58]. Lastly, the increased intensity of the band near 450 cm^−1^ corresponds to the presence of Si–O–Si bending (or rocking) vibrations typical of silica and silicate materials [59].

### 3.2. Thermal Properties

TGA was employed to primarily assess the thermal stability of the reinforced samples and, subsequently, their dispersion efficacy. To determine the filler content, the samples were heated to 600 °C, well above the decomposition temperature of PVB. The residual mass was determined after each run, composed of the silicate dispersion, and minimal char residue from the PVBR-B matrix. Since the inorganic filler is thermally stable under these conditions, the experimental filler content can be estimated by subtracting the residual mass of the pure polymer from the total residue of the composite sample (Equation (4)). Multiple measurements enabled a more reliable assessment of the uniformity of filler dispersion throughout the composite.

(4) Residue of composite=Residue of matrix+Residue of Filler

**Figure 5:**
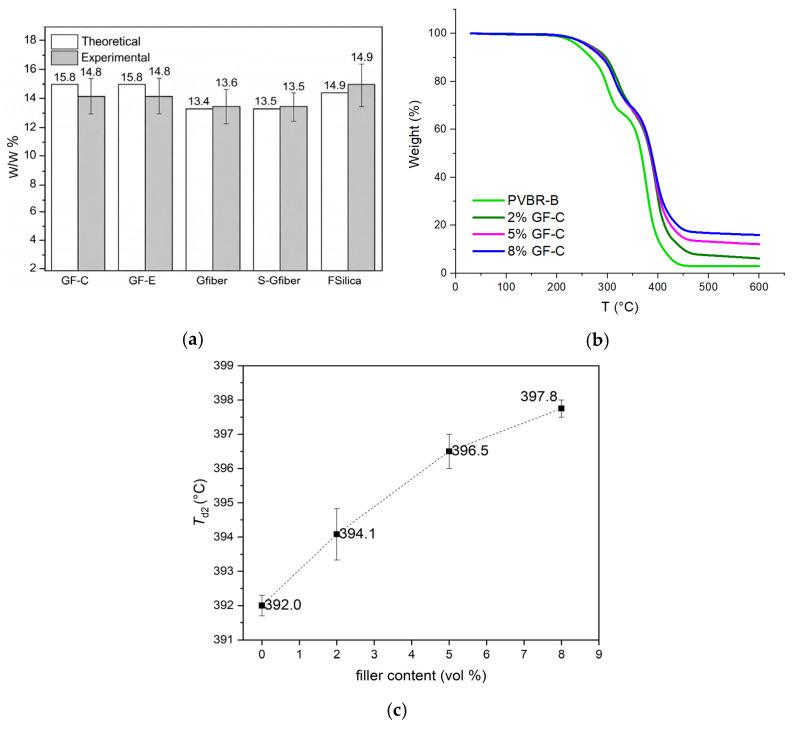
(**a**) Theoretical concentration compared to the experimental concentration estimated via TGA at 8 vol% content for composites (GF-C, GF-E, Gfiber, S-Gfiber, F-Silica); (**b**) indicative TGA graph for increasing filler content (2, 5, 8 vol%) of GF-C reinforcement; (**c**) *T*_d2_ with increasing fumed silica content.

Equation (4) describes the composition of residual mass for each sample. Inorganic residue was in accordance with the original synthesis composition and with a low deviation (1.2–1.9%), representing that the reinforcement has been adequately dispersed within the PVB matrix (Table S1). Vol% contents of composites were converted to theoretical w/w% using the corresponding densities (Table S1). The extracted theoretical inorganic filler concentration was then compared with the experimental values obtained from TGA (Figure 5a). The experimentally determined inorganic residue values obtained by TGA are in close agreement with the theoretical values calculated from the formulations for all composite systems. The small deviations observed fall within experimental uncertainty and indicate good reproducibility across replicate measurements. This agreement confirms that the targeted filler loadings were effectively incorporated during melt mixing and that the resulting composites are compositionally uniform at the bulk scale. In addition, thermographs were plotted, displaying the tendency of increasing residue with increasing concentration of each dispersion (Figure S1). The satisfying incorporation of the filler demonstrates the ability to implement these dispersions in industrial-scale melt mixing processes.

Also, the plasticizer concentration was calculated at 31.0 ± 0.9% for PVBR-B, which is considered within the typical range of plasticization for a plasticized PVB grade used commercially for mostly laminated glass applications [4]. Therefore, the interactions of plasticized PVB with silicate dispersions in this paper are beneficial for the exploitation or upcycling of commercial-recycled PVB grades.

Concerning the thermal stability of composites, there were no significant alterations concerning the *T*_d1_ and *T*_d2_ of the samples apart from the case of fumed silica (Table S1). In addition, the associated standard deviation values for all samples were considered low (reaching up to ±1.7 °C), showing clear trends. At 2 vol% content of fumed silica, an increase of ca. 30 °C in *T*_d1_ was observed, suggesting that silica can retard the evaporation of plasticizer. This is attributed to more efficient bonding of the polymeric matrix with the reinforcement, therefore suppressing evaporation. For 5 and 8 vol% content, the plasticizer can slow volatilization, resulting in a smoother evaporation without a peak temperature (Figure S1e). This indicates a form of thermal stabilization, suggesting that a broader and higher temperature range is required for the plasticizer to evaporate, possibly due to the Si–O–C linkage network, which affects the chemical structure and results in a more rigid polymeric matrix.

Regarding PVB decomposition temperature (*T*_d2_), addition of glass dispersions (glass flakes or glass fibers) did not affect the decomposition temperature, remaining constant at ca. 390 °C for most of the cases (Table S1). However, the use of fumed silica increased this temperature up to 7 °C at 8 vol% due to restricted chain mobility, proving that it can have a barrier effect in the decomposition of PVB (Figure 5c).

### 3.3. Mechanical Properties

**Figure 6:**
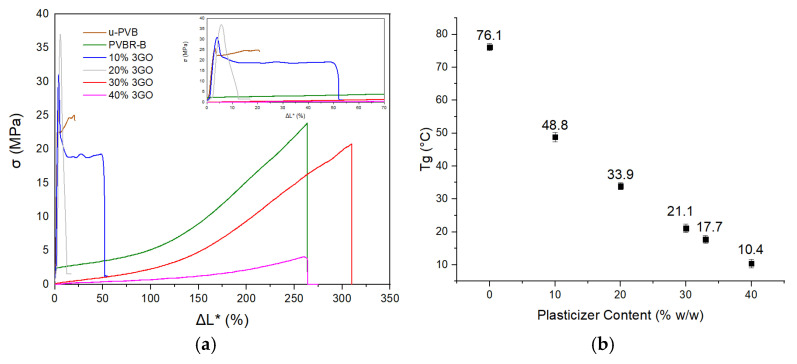
Influence of increasing plasticizer concentrations (0, 10, 20, 30, 40% *w*/*w*) (**a**) on the mechanical behavior of PVB, and (**b**) on the *T*_g_ of the samples.

Tensile and impact performance of the composites were analyzed, emphasizing the role of filler morphology and interfacial adhesion to the mechanical performance. The tensile behavior was initially examined only for plasticized PVB samples, evaluating how the varying plasticizer concentration (10, 20, 30, and 40% *w*/*w* 3GO) influences stiffness, flexibility, and overall mechanical performance before the incorporation of fillers into the plasticized PVBR-B. According to the stress-normalized displacement diagram (Figure 6a), neat unplasticized PVB (u-PVB) presented a short elastic region (up to 5% displacement), followed by plastic deformation with a small displacement up to 25%. The addition of the plasticizer resulted in reducing *T*_g_ (Figure 6b) enhancing the molecular mobility of the PVB chains, consequently altering the mechanical behavior of PVB. The material transitioned from a brittle behavior with minimal elongation, indicative of a glassy state to a viscoelastic or rubbery behavior [60]. This state is characterized by a significant increase in displacement (up to 320% for 30% plasticizer), reduction in tensile strength (up to 4.9 MPa for 40% plasticizer) and a decrease in stiffness (from 1.95 GPa to 1.04 MPa). Plasticizer can effectively weaken the intermolecular forces and lead to a significant *T*_g_ reduction from 76 °C to 10 °C (Figure 6b) [2].

PVBR-B used in this study of composites exhibited a similar behavior to PVB plasticized with 30% *w*/*w* 3GO and *T*_g_ = 17.7 °C. It exhibited a rubber-like state with only elastic behavior and reversible deformations, returning to its original length, proving that the polymer chains presented enough mobility to reorient and uncoil under tension. Deviations of PVBR-B mechanical behavior compared to the 30% plasticized PVB sample with 3GO could be attributed to different molecular weight or vinyl alcohol content. PVBR-B values for tensile strength (20 MPa) and stiffness (4 MPa) were in the expected range [14,19,42]. Thus, it was used as a representative polymeric matrix, as its composition closely reflects the plasticizer levels of a standard commercial and recycled PVB grades, making its interaction with silicate dispersions more indicative of realistic PVB composite conditions [36].

**Figure 7:**
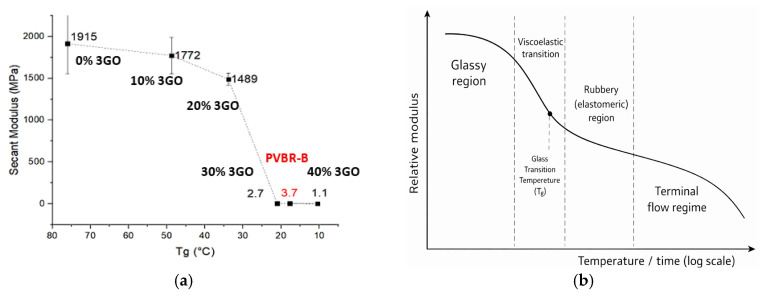
(**a**) *T*_g_ values for plasticized samples (10, 20, 30, 40%) derived from DSC plotted against the corresponding Secant Modulus (E) values; (**b**) the elastic modulus dependence on (logarithmic) time and temperature for a typical thermoplastic polymer. The dotted vertical lines schematically indicate the boundaries between the glassy, viscoelastic transition, rubbery, and terminal flow regimes [60].

The low *T*_g_ values ensure that PVB films remain in the rubbery state under stress conditions in laminated glass. In this state, the films exhibit high displacement at fracture, essential for their applications. The stress-normalized displacement curves exhibited markedly different behaviors depending on the plasticizer concentration. Figure 7a shows that adding plasticizer softens the matrix and reduces stiffness, consistent with the transition of plasticized PVB [10]. Higher variability of Secant Modulus values observed at low plasticizer concentrations (0–20% *w*/*w*) reflects the increased sensitivity of glassy PVB to small experimental variations, such as specimen thickness. In contrast, variability becomes negligible at higher plasticizer concentrations. Comparison of Figure 7a with Figure 7b shows that PVBR-B belongs to the rubbery plateau close to the viscoelastic transition. A plasticizer concentration of 20–30% *w*/*w* places the material within its viscoelastic transition region, under the controlled ambient conditions of 21 ± 2 °C, while increasing the plasticizer more to the rubbery plateau. Excess plasticizer can shift the material toward more viscous flow at room temperature or else the polymeric matrix can reject its absorption.

The state of the polymeric matrix can significantly influence the interfacial behavior with reinforcement and eventually the mechanical properties of the composite. PVBR-B is in a rubbery–viscoelastic state, exhibiting a more fluid-like behavior that may result in weaker or less controlled interactions with the reinforcement. Under quasi-static tensile loading, plasticized PVB exhibits a nonlinear, largely recoverable deformation that is commonly described as hyperelastic-like behavior. As the strain rate increases, the ductility of PVB decreases and its mechanical response progressively transitions toward elastic–plastic behavior. At room temperature, plasticized PVB displays a predominantly viscoelastic–rubbery response, since its glass transition temperature lies close to room temperature. Consequently, its stress–strain behavior is nonlinear and strongly dependent on both temperature and strain rate [19,20,21,23,43]. In this study, all mechanical tests in PVB or PVB composites were performed at a fixed displacement rate and at room temperature to ensure consistent and meaningful comparison between composite formulations. Additionally, plasticized PVB demonstrates an energy-absorbing mechanical response, indicating that stress can be more effectively transferred to the polymeric matrix rather than reinforcement. When the plasticizer concentration is moderate, adhesion between the matrix and the reinforcement is considered adequate or can even be improved due to enhanced wetting. However, at high plasticizer concentrations, adhesion typically decreases, leading to reduced interfacial strength and potential debonding under stress or thermal cycling. The plasticizer concentration plays a pivotal role in determining the mechanical behavior of the composites because the load transfer from the glass to the matrix diminishes as the matrix becomes highly plasticized. In addition, plasticizers can interfere with the available -OH hydroxyl content for chemical interactions, therefore altering the interactions [61]. As a result, the findings of this study are directly applicable only to PVB samples with similar plasticizer concentrations.

**Figure 8:**
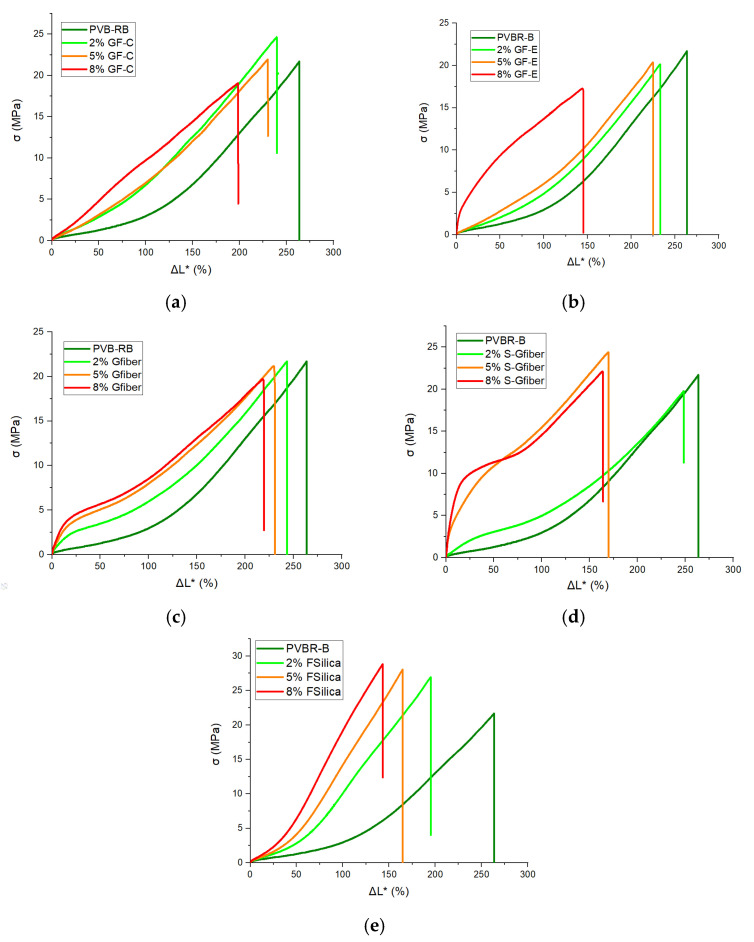
Stress-normalized displacement graph at various filler contents (2, 5, 8 vol%) for (**a**) GFC; (**b**) GF-E; (**c**) Gfiber; (**d**) S-Gfiber; (**e**) F-Silica.

Composites were prepared based on vol% content, since stress and strain are distributed through the volume of the material, therefore the comparison between the different samples should be according to that [47]. Figure 8 presents the mechanical behavior for each different reinforcement (GF-C, GF-E, GFiber, S-GFiber, F-Silica) at 2, 5, and 8 vol%.

GF-C reinforcement disrupted the elastic (nonlinear) behavior, moving to a straight line in the stress-normalized displacement diagram (Figure 8b). The addition of glass flakes of higher aspect ratio (GF-E) significantly impacts the behavior of the composite only at 8 vol% (Figure 8b). This could be due to ‘flakes to flakes’ interactions and possible agglomeration leading to stress concentration sites and premature failure, reducing displacement. The addition of glass fibers (Figure 8c) altered the mechanical behavior of PVB, exhibiting a yield point at almost 25% displacement after this point changed to plastic deformation. The introduction of glass fibers into the system interfered with the reversible deformation of the material limiting the plasticizer action as can be seen in the pictures (Figure S2). Treatment of glass fibers with silane solution significantly changed the overall behavior of the composite. Additionally, a yield point at 25% displacement is also observed in both no-treated and treated cases. The 2 vol% S-Gfiber behaved similarly to 2 vol% Gfiber, but 5% and 8 vol% S-Gfiber presented a different behavior. Composites with fumed silica presented significantly increased tensile strength while maintaining similar reversible hyperelastic behavior and deformation, same as reference PVBR-B.

The different behavior of the stress–normalized displacement curves can be interpreted in terms of the constitutive response of plasticized PVB. Under the quasi-static conditions plasticized PVB (PVBR-B) exhibits a rubbery, nonlinear response with large recoverable strains, which is often described as hyperelastic. In contrast, the appearance of a yield point and subsequent irreversible deformation in selected formulations, notably in the fiber and flake filled systems, indicates a transition toward elastic–plastic behavior, due to localized stress concentrations [21]. Transitions to elastic–plastic behavior are expected to become more pronounced at higher strain rates [20,24,25].

**Figure 9:**
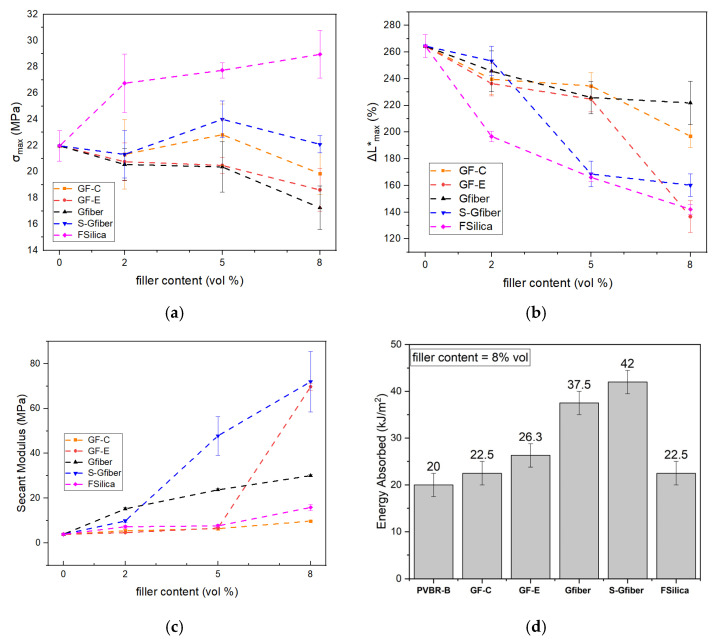
Graphs of filler content for all composite formulations (GF-C, GF-E, GFiber, S-GFiber, F-Silica) vs. (**a**) maximum stress (σ_max_); (**b**) maximum normalized displacement (ΔL*_max_); (**c**) Secant Modulus; as a function of increasing filler content, (**d**) impact resistance of PVB composites at 8 vol% filler content.

Focusing on tensile stress at fracture (*σ*_max_), normalized displacement at fracture (ΔL*_max_) and Secant Modulus (*E*), a deeper understanding of the interface can be extracted (Figure 9a–c).

Previous studies have reported that the incorporation of waste flat glass can enhance the tensile strength of PVB up to an optimal content, beyond which the strength declines [42]. Furthermore, smaller glass beads are known to induce lower interfacial stress concentrations, thereby increasing the failure stress [40]. Accordingly, GF-C, which consists of small glass flakes, appears to follow the same trend, exhibiting a minor increase in tensile strength (23 MPa) at 5 vol%, while further addition of the reinforcement leads to a decrease in tensile strength. In contrast, the addition of GF-E, which consists of larger flakes, tends to promote agglomeration, thereby reducing the tensile strength (19 MPa at 8 vol%). Addition of glass fiber (Gfiber) mainly decreased tensile strength, while treated glass fibers (S-Gfiber) had improved compatibility, chemical interaction and stress transfer at the interface [41,54]. The tensile strength reached its maximum value at 5 vol% with further addition of filler (S-Gfiber) disrupting the system’s homogeneity, thus leading to a decrease in tensile strength. Fumed silica can exhibit strong chemical interactions with PVB through hydrogen bonding between its surface –SiOH groups and the –OH groups of PVB. The strong interfacial interactions and high specific surface area of fumed silica promote the formation of a reinforcing network capable of efficiently transferring stress and enhancing the overall mechanical performance of the composite. This effect is clearly reflected in the tensile strength of PVB, which increases systematically with rising fumed silica content, reaching up to 29 MPa at 8 vol% (Figure 9).

Normalized displacement at fracture shows an inverse relationship with tensile strength, with lower values generally corresponding to stronger interfacial interactions (Figure 9a,b). GF-C retained high displacement at fracture values, suggesting that the small-sized flakes did not interfere with the polymer’s flexibility. In contrast, GF-E, when incorporated at a higher contents (8 vol%), likely led to agglomeration, reduced and disruption of the polymeric network, resulting in a significant decrease (to 130%). Glass fibers had minimal impact on displacement at fracture (230% at 8 vol%), allowing for easier material deformation, indicating low chemical interaction with the polymer matrix. Yet, treated glass fibers (S-Gfiber) exhibited markedly different behavior, as the increased chemical interaction led to stronger bonding between the fibers and the polymer matrix. Fumed silica, which exhibited the highest tensile strength along with the lowest displacement at fracture (140% at 8 vol%), showed that the formation of hydrogen-bonded, three-dimensional networks strongly restricts the mobility of polymer chains and counteracts the plasticizing effect. Silica-based reinforcements, capable of bonding with PVB through silanol groups, appear to exert the effect opposite to that of plasticizers. Their chemical interactions with the matrix highlight the contrasting mechanisms of plasticization and silicate reinforcement.

Figure 9c illustrates the variation in the Secant Modulus with increasing filler content. GF-C showed minimal increase—practically remaining constant—in stiffness, possibly due to low aspect ratio and random orientation of the reinforcement, resulting in an isotropic but lower modulus. On the other hand, GF-E, exhibits a higher aspect ratio, potentially enables more efficient stress transfer and thus provides stronger reinforcement. This effect was observed mainly at 8 vol% (E = 70 MPa), where partial flake alignment may have been more pronounced. Glass fibers significantly increased modulus in all volume contents, as their long, and thin geometry promotes mechanical interlocking, enabling crack bridging, improved load distribution, and enhanced barrier properties. S-Gfiber samples exhibited enhanced interfacial bonding, leading to an increase in Secant Modulus up to 70 MPa. This improvement can be attributed to the stronger interaction with the polymer matrix, which reinforced the material along the uniaxial loading direction. Although fumed silica did not increase the Secant Modulus, it contributed to enhanced tensile strength. This suggests that any potential increase in stiffness from the filler may have been covered by the large modulus drop induced by plasticization. In summary, plasticized PVB typically exhibits a glass transition temperature (*T*_g_) close to room temperature; therefore, in the vicinity of *T*_g_, the matrix behaves in a rubbery manner and becomes less sensitive to rigid particles. Consequently, temperature and environmental conditions can further influence the mechanical response of the composite.

Classical micromechanical models such as the rule of mixtures, Voigt iso-strain, Guth–Gold, or Halpin–Tsai equations are derived under assumptions of linear elasticity and Young’s modulus. In the present work, a Secant Modulus determined at finite strain was estimated, which precludes the direct application of these models. Furthermore, the presence of a plasticizer in the PVB matrix introduces additional complexity by modifying both the effective matrix modulus and the matrix–filler interactions. Consequently, prediction of the composite modulus would require a revised theoretical framework accounting for plasticizer and interfacial effects [62].

The impact resistance of PVB composites was evaluated using Izod impact testing to assess their ability to absorb energy under loading in the presence of silica-based reinforcements. Specifically, PVBR-B exhibited an absorbed energy of approximately 20 ± 2 kJ/m^2^, while the GF-C and fumed silica composites showed comparable values of around 22.5 ± 2 kJ/m^2^ (Figure 9d). This indicates that the reinforcing mechanism observed in tensile testing specifically for fumed silica composites does not similarly contribute to significantly improved impact strength behavior. On the other hand, glass flakes (GF-E) with higher aspect ratios and chopped glass fibers (Gfiber) significantly enhanced the energy absorption capacity of the composites to 26.3 and 37.5 kJ/m^2^, respectively, indicating effective stress redistribution and crack-deflection mechanisms during impact (Figure 9d). This could be associated with their ability to bridge microcracks or absorb energy by themselves, thereby increasing the energy required for fracture. Moreover, the improved interfacial interactions due to silane treatment further enhanced energy transfer efficiency and impact performance. This effect was noticed for S-Gfiber, which exhibited a markedly higher absorbed energy (42 kJ/m^2^) compared to the composite reinforced with untreated glass fibers.

Overall, while plasticizers increase chain mobility, making PVB more flexible enhancing its energy absorption capacity, the incorporation of specific reinforcements can further augment the ability of plasticized PVB to absorb energy. Plasticized PVB exhibits a rubbery–viscoelastic behavior at room temperature, characterized by high chain mobility and large reversible deformation. The incorporation of silica-based fillers introduced regions of restricted segmental motion near the filler–matrix interface due to the abundance of surface silanol groups promoting extensive hydrogen bonding with the hydroxyl groups of PVB making physical crosslink. This microstructure explains the mechanical behavior which exhibited increase in tensile strength and reduction in displacement at fracture. However, hyperelastic behavior of PVB was not disrupted and no alteration in impact resistance was mentioned. Conversely, glass flakes and fibers, particularly at higher aspect ratios, acted as load-bearing inclusions that disrupted the hyperelastic behavior of PVB, enhanced stiffness and impact resistance. They are expected to have a less pronounced effect on the chain mobility of the polymer and they also demonstrated an ability to absorb impact resistance. The latter composites could be utilized in advanced laminated glass to further enhance energy absorption, when high transparency is not essential.

### 3.4. Water Absorption–Hygroscopicity

Moisture uptake and diffusion behavior of the PVB composites were investigated to assess their durability under humid conditions. Moisture content is critical for the quality of plasticized PVB, strongly influencing the lamination process and the adhesion to the glass sheet. PVB is considered moderately hygroscopic and susceptible to hydrolysis to its ester groups [46,48]. Therefore, the various PVB composites were evaluated for their barrier efficiency against water absorption. According to literature, the optimum moisture content of PVB is 0.45% ± 0.07% demonstrating the necessity for low moisture content [43]. From a water content of 1% the adhesive bond can be strongly disrupted, and raising above 2% potentially affects the transparency of the film, resulting in milky or cloudy appearance [12,63].

**Figure 10:**
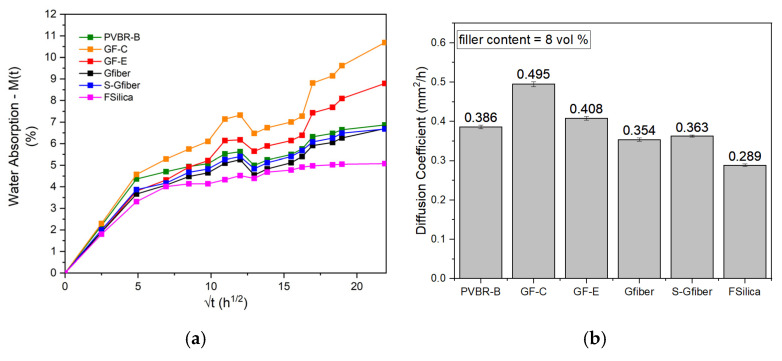
All tested composites (GF-C, GF-E, GFiber, S-GFiber, F-Silica) at 8 vol% content regarding (**a**) water absorption (%) vs. square root of time ($\sqrt{h}$); (**b**) Diffusion Coefficient (mm^2^/h).

Figure 10a displays the water absorption behavior of plasticized (PVBR-B) and composite PVB samples. During the initial stage of exposure, water uptake occurred rapidly for all specimens and gradually slowed until stabilization, which was achieved roughly at 20 days. Composites with glass flakes GF-C and GF-E presented the highest absorbed water content of 11 and 8% respectively, even higher than the reference plasticized PVB (7%). This could be due to the formation of voids occurring between the interface of PVB and the silicate dispersion. Considering the smaller size of GF-C, void formation is more likely, due to a higher number of potential discontinuities occurring within the material compared to GF-E, which consists of larger particles and exhibited better performance. Furthermore, the flat surface and morphology of the flakes may also influence water absorption behavior by promoting looser interfacial interactions and thereby allowing greater water penetration. Glass fibers (Gfiber) and functionalized glass fibers (S-Gfibers) behaved slightly better than plasticized PVB, acting potentially as a water barrier. The composites containing fumed silica exhibited significantly greater resistance to water absorption and minimum moisture uptake (M = 4.5) compared to the neat PVB. This behavior can be attributed to the interaction between fumed silica and the vinyl alcohol groups of PVB, which reduces the availability of hydrophilic sites and thus limits moisture absorption. For these systems, the Fickian diffusion coefficients (D) were calculated as follows: PVBR-B: 0.386 ± 0.005 mm^2^/h, and for the composites of 8 vol% filler content GF-C: 0.495 ± 0.006, GF-E: 0.408 ± 0.005, Gfiber: 0.354 ± 0.004, S-Gfiber: 0.363 ± 0.003, and F-Silica: 0.289 ± 0.003 mm^2^/h (Figure 10b). The small standard deviations observed for all formulations suggest homogeneous filler dispersion and consistent water absorption behavior across specimens.

Water immersion tests were employed to simulate the long-term exposure of PVB composite materials to humid environmental conditions, similar to those encountered in building façades or protective coatings, to assess the efficiency of the developed formulations. Since both fumed silica and glass fibers improved moisture resistance, they can be effectively utilized as reinforcements against humidity-induced degradation [64]. The reduced moisture absorption observed can be correlated with enhanced interfacial adhesion between the polymer matrix and the glass phase. Adding to that, water absorption and diffusion testing serve as valuable tools for evaluating the durability and adhesion performance of protective polymer coatings and enhanced interlayer material for exterior applications (e.g., windshields, facades, PV modules) [65,66].

### 3.5. Melt Rheology-Processability

**Figure 11:**
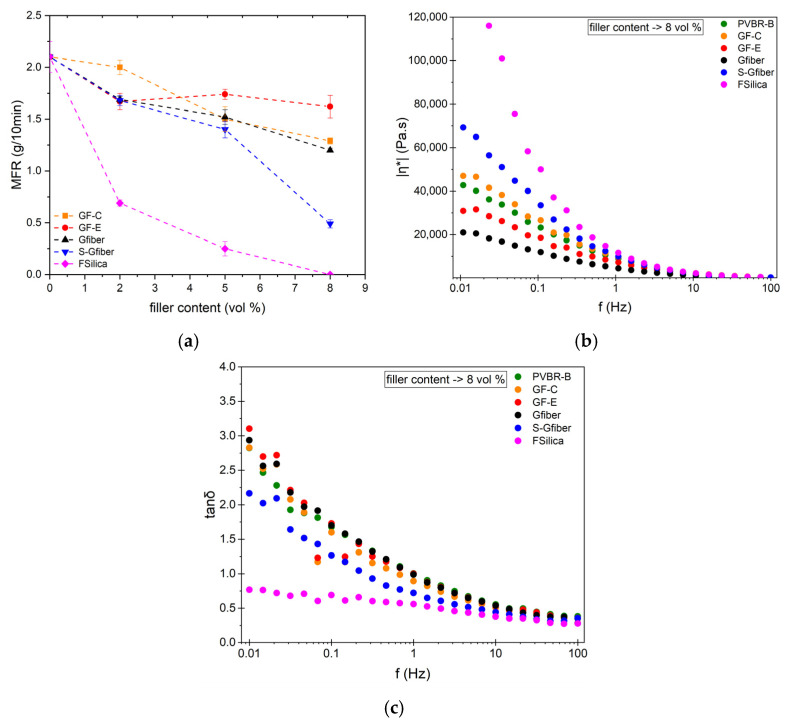
All tested composites (GF-C, GF-E, Gfiber, S-GFiber, F-Silica) regarding (**a**) MFR values as a function of various contents vol% (2, 5, 8%); (**b**) complex viscosity as a function of frequency at 8 vol%; (**c**) tan δ as a function of frequency at 8 vol%.

The melt rheology of the various composite formulations was evaluated through melt flow rates (MFRs) and dynamic oscillatory measurements to evaluate their processability under industrially relevant conditions. MFR serves as an indicator of their accordance with the existing processing conditions of PVB. Figure 11a presents MFR results from the initial formulation without reinforcements, yielding a value of 2.1 g/10 min, which is within the range of a typical plasticized PVB sample [36]. All composite formulations exhibited an increase in melt viscosity, with viscosity further rising as the filler content increased. At 8 vol%, GF-C reinforcement reduced the melt flow rate (MFR) to 1.4 g/10 min, similarly to GF-E reinforcement (1.6 g/10 min), suggesting that, within the investigated range, differences in flakes size does not severely affect melt flow. Incorporation of glass fibers (Gfiber) moderately decreased MFR, showing a reduction comparable to that observed for glass flakes. The notably low MFR (0.4 g/10 min) of S-Gfiber composite at 8% volume content may be associated with improved interfacial interactions, consistent with the observed increase in the Secant Modulus (E). Fumed silica is unsuitable for contents close to 8 vol%, as the melt viscosity increases significantly, obstructing its processability. The particles of fumed silica tend to form a network through hydrogen bonding of surface –SiOH (silanol) groups within the polymer melt, which restricts polymer chain mobility, and therefore, no melt flow was observed at 8 vol%.

While MFR provides a single-point measure of melt flow under a fixed load and temperature, it does not capture the frequency-dependent viscoelastic behavior of the material. Therefore, oscillatory melt rheology was also employed at a temperature representative of industrial PVB processing (*T* = 150 °C) to assess the deformation response over a broad range of frequencies, enabling a more comprehensive evaluation of melt processability and network formation [65]. All formulations exhibited pronounced shear-thinning behavior, with elevated complex viscosity values at low frequencies that progressively decrease with increasing frequency (Figure 11b). The strong increase in low-frequency |η*| with filler content, particularly for fumed silica reinforced PVB, indicates the development of an elastic network that resists long-time deformation. The corresponding tan δ graph (Figure 11c) displays a monotonic decrease with increasing frequency for all compositions, without the appearance of a relaxation peak. Silica-filled system exhibits the lowest tan δ values (tan δ < 1) across the entire frequency range, reflecting reduced viscous dissipation and a higher effective plateau modulus. Τan δ values below unity over the entire frequency range indicate a solid-like response [67,68]. This behavior is attributed to the formation of a silica network that restricts polymer chain mobility, suppresses viscous dissipation and limiting flow even at long times.

The extent of viscosity change depends strongly on the filler type, morphology, and its chemical interactions. Therefore, the filler content should be selected to ensure sufficient reinforcing effect while maintaining processability. Otherwise, increased reinforcement content will require higher processing temperatures to overcome excessive viscosity, potentially leading to thermal degradation of the material and/or plasticizer evaporation.

## 4. Conclusions

This study systematically examined plasticized PVB composites reinforced with silicate or silica fillers of different morphologies (glass flakes, glass fibers, and fumed silica) at 2–8 vol% under melt mixing conditions. Transparency measurements revealed that fumed silica maintained the same transparency levels as neat PVB (~82%), while the addition of fibers and flakes caused a significant reduction, especially in silane-treated fibers (~60%). Thermal characterization showed no substantial changes in the thermal behavior of the composites, except for fumed silica which exhibited significantly increased thermal stability with increases of up to ~30 °C in plasticizer evaporation temperature and ~7 °C in PVB decomposition temperature. Fumed silica exhibited the strongest reinforcing effect in tensile tests, increasing tensile strength up to 29 MPa at 8 vol% and reducing displacement at fracture to ~140%. High-aspect-ratio flakes and silane-treated glass fibers were most effective in increasing composite Secant Modulus, with values reaching approximately 70 MPa at 8 vol%. Also, mechanical testing was conducted at a single displacement rate at room temperature, while PVB exhibits a strain-rate- and temperature-dependent behavior. In addition, high-aspect-ratio glass flakes and chopped glass fibers significantly enhanced impact resistance, reaching 37.5 kJ/m^2^ and 42 kJ/m^2^ for untreated and silane-treated fibers, respectively, albeit the reduced transparency. This suggests that glass fibers could be beneficial for interlayer applications where transparency is not critical, offering improved impact resistance in advanced laminated glass composites. Long-term durability aspects, such as aging and fatigue were not investigated for the developed composites and may affect the mechanical and interfacial properties reported herein. Regarding water absorption, composites with glass flakes (GF-C and GF-E) exhibited increased water uptake (8–11%), likely due to void formation around fibers, whereas fumed silica significantly reduced water absorption (4.5%), indicating its potential use for exterior applications. From an industrial standpoint of melt processing, fumed silica in comparison to other fillers severely decreased melt flow at all volume contents, hindering processability beyond 5 vol%. Similarly, silane-treated glass fibers affected processability above 8 vol%, while other reinforcements had a negligible influence. Overall, this paper provides an insight into tailoring PVB composites with a polymeric matrix of specific plasticizer concentration for the upcycling or valorization of secondary PVB grades, and sheds light on the interactions of PVB with various silicate/silica reinforcements.

## Figures and Tables

**Table 1:** Description of incorporated fillers as provided by the suppliers.

Reinforcement	Commercial Name	Morphology	Chemical Composition	Specifications	Supplier
GF-C	GF003	Flake, low aspect ratio (AR ≃ 10)	ECR-glass	*d*_50_ = 27–32 μm thickness = 2.3–3.3 μm	GlassFlakes Co., Ltd. (Leeds, UK)
GF-E	750MC	Flake, high aspect ratio (AR ≃ 20)	C-glass	*d*_50_ = 90 ± 20 μm thickness = 5 ± 2 μm	GlassFlakes Co., Ltd. (Leeds, UK)
Gfiber	n.d.	Chopped Fiber	E-glass	L = 10–25 mm thickness = 5–15 μm	NEOTEX S.A. (Athens, Greece)
FSilica	Aerosil 200	Nanoparticles	Fumed silica	*d*_50_ = 7–20 nm Specific surface area 200 m^2^/g	NEOTEX S.A. (Athens, Greece)

## Data Availability

The original contributions presented in this study are included in the article and Supplementary Material. Further inquiries can be directed to the corresponding authors.

## Supplementary Materials

The following supporting information can be downloaded at https://www.mdpi.com/article/10.3390/polym18040476/s1, Table S1: Filer content (vol%), theoretical inorganic residue (% *w*/*w*) and *T*_d1_, *T*_d2_ & Experimental Inorganic filler content as they were extracted after the evaluation of TGA mass loss curves; Figure S1: TGA mass loss curves for all the different reinforcements or all the different filler contents (2, 5, 8 vol%) tested (a) GF-C; (b) GF-E; (c) Gfiber; (d) S-Gfiber; (e) F-Silica; Figure S2: (a) Tensile specimens before measurement; (b) Tensile specimens from composite of GF-C 8 vol% after test (showed only elastic deformation); (c); Tensile specimens from composite filled with 8 vol% glass fibers (showed plastic deformation).
