# Supplementary material for: Poly(vinyl butyral) Composites with Different Silicate or Silica Dispersions
Source: Polymers (Basel). 2026 Feb 13;18(4):476. doi: 10.3390/polym18040476 (PMC12944061; doi:10.3390/polym18040476)
Supplement: Supplementary file 1 [file polymers-18-00476-s001.zip › polymers-4087032-supplementary.pdf]

SUPPLEMENTARY INFORMATION

Table S1: Filer content (vol %), theoretical inorganic residue (w/w %) and T_{d1}, T_{d2} & Experimental Inorganic filler content as they were extracted after the evaluation of TGA mass loss curves.

	Filler Content (vol %)	T_{d1} (plasticizers evaporation)	T_{d2} (PVB decomposition)	Experimental Inorganic filler content extracted from TGA (Eq.3) (w/w %)	Theoretical Inorganic Residue (w/w%)
PVBR-B	0	310.2 ± 0.9	392.0 ± 0.3	1.4 ± 1.4	0
GF-C (d=2.6g/mL)	2	322.5 ± 1.1	391.3 ± 1.6	3.9 ± 1.9	4.7
	5	313.83 ± 1.3	392.3 ± 1.6	9.8 ± 1.8	11.5
	8	310.3 ± 0.9	390.0 ± 1.1	14.8 ± 1.4	15.8
GF-E (d=2.6 g/mL)	2	320.8 ± 0.5	392.0 ± 1.1	5.1 ± 1.4	4.7
	5	323.6 ± 0.6	392.5 ± 1.8	9.4 ± 1.8	11.5
	8	313.0 ± 1.1	390 ± 3.6	14.8 ± 1.4	15.8
Gfiber (d= 2.4 g/mL)	2	310.6 ± 1.3	390.5 ± 1.7	5.3 ± 1.5	3.9
	4	315.8 ± 0.7	390.6 ± 0.5	9.9 ± 1.4	9.8
	8	319.5 ± 0.4	391.5 ± 1.3	14.6 ± 1.4	13.4
Sgfiber (d=2.4 g/mL)	2	323 ± 0.2	389.3 ± 0.4	6.2 ± 1.3	3.9
	5	310 ± 0.4	388.0 ± 0.7	11.5 ± 1.4	9.8
	8	325 ± 1.5	389.5 ± 0.9	14.5 ± 1.2	13.4
Fsilica (d= 2.2 g/mL by literature)	2	345 ± 1.6	394.1 ± 0.8	5.2 ± 1.4	4.3
	5	No peak	396.5 ± 0.5	9.9 ± 1.4	10.5
	8	No peak	397.8 ± 0.3	14.9 ± 1.5	14.3

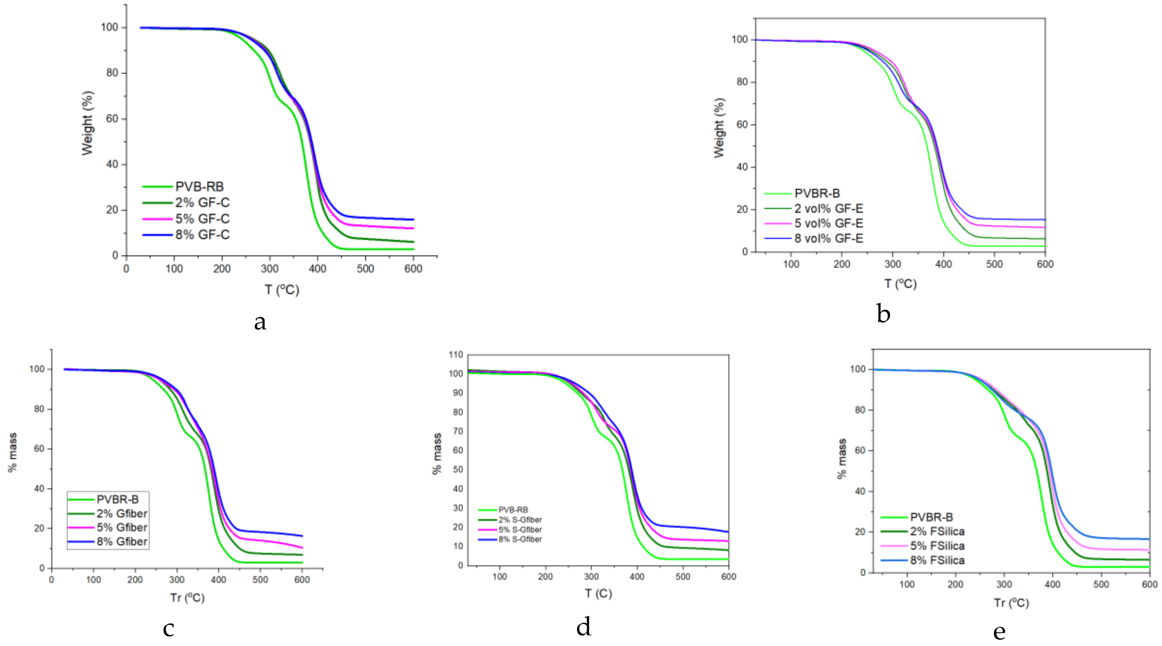

Figure S1: TGA mass loss curves for all the different reinforcements or all the different filler concentrations (2, 5, 8 vol %) tested (**a**) GF-C; (**b**) GF-E; (**c**) Gfiber; (**d**) S-Gfiber; (**e**) FSilica;

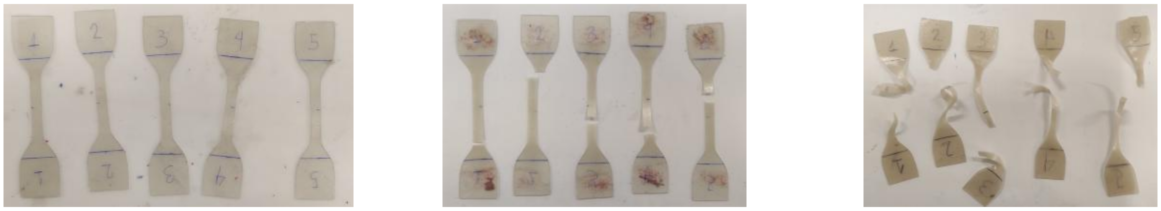

a **b** **c**

Figure S2: (**a**) Tensile specimens before measurement; (**b**) Tensile specimens from composite of GF-C 8 vol % after test (showed only elastic deformation); (**c**); Tensile specimens from composite filled with 8 vol % glass fibers (showed plastic deformation).
